# Supplementary material for: Practices and outcomes of responsive caregiving on child neurodevelopment and mental health across diverse global populations: a scoping review protocol
Source: BMJ Open. 2024 Apr 3;14(4):e078712. doi: 10.1136/bmjopen-2023-078712 (PMC7615939; doi:10.1136/bmjopen-2023-078712)
Supplement: Supplementary file 1 [file bmjopen-2023-078712.draft_revisions.pdf]

BMJ Open is committed to open peer review. As part of this commitment we make the peer review history of every article we publish publicly available.

When an article is published we post the peer reviewers' comments and the authors' responses online. We also post the versions of the paper that were used during peer review. These are the versions that the peer review comments apply to.

The versions of the paper that follow are the versions that were submitted during the peer review process. They are not the versions of record or the final published versions. They should not be cited or distributed as the published version of this manuscript.

BMJ Open is an open access journal and the full, final, typeset and author-corrected version of record of the manuscript is available on our site with no access controls, subscription charges or pay-per-view fees (http://bmjopen.bmj.com).

If you have any questions on BMJ Open's open peer review process please email info.bmjopen@bmj.com

BMJ Open

Practices and outcomes of responsive caregiving on child neurodevelopment and mental health across diverse global populations: A scoping review protocol

Journal:	*BMJ Open*
Manuscript ID	bmjopen-2023-078712
Article Type:	Protocol
Date Submitted by the Author:	09-Aug-2023

Complete List of Authors:	Lobo, Eunice; Indian Institute of Public Health - Bengaluru, Public Health Foundation of India; Institute of Public Health Bengaluru<br>Mahapatra, Sandeep; Evalueserve, Bangalore, India<br>Babu, Giridhara R; Indian Institute of Public Health - Bengaluru, Public Health Foundation of India; DBT/Wellcome Trust India Alliance<br>van Schayck, Onno; Maastricht University Care and Public Health Research Institute<br>Prashanth, NS; Institute of Public Health Bengaluru<br>Mukherjee, Debarati; Indian Institute of Public Health - Bengaluru, Public Health Foundation of India

Keywords:	Community child health < PAEDIATRICS, Developmental neurology & neurodisability < PAEDIATRICS, PUBLIC HEALTH

SCHOLARONE™
Manuscripts

Title: Practices and outcomes of responsive caregiving on child neurodevelopment and mental health across diverse global populations: A scoping review protocol

Authors: Eunice Lobo[1,2,3], Sandeep Mahapatra[4], Giridhara R. Babu[1,5] Onno C P van Schayck[3], Prashanth Nuggehalli Srinivas[2], Debarati Mukherjee[1]

Affiliations:

1. Indian Institute of Public Health - Bengaluru, Public Health Foundation of India, India
2. Institute of Public Health, Bengaluru, India
3. Care and Public Health Research Institute, Maastricht University, The Netherlands
4. Evalueserve, Bengaluru, India
5. DBT-Wellcome Trust India Alliance, Hyderabad, India

Corresponding author: Dr. Debarati Mukherjee

Address: Indian Institute of Public Health-Bengaluru, Public Health Foundation of India (PHFI), Besides Leprosy Hospital, 1st Cross, Magadi Road, Bengaluru, India – 560023

Email: debarati.mukherjee@phfi.org

For peer review only

Abstract

Introduction: Responsive caregiving (RC) leads to positive outcomes in children, including secure attachment with caregivers, emotional regulation, positive social interactions, and cognitive development among others. Through our scoping review, we aim to summarize the practices and outcomes of RC in diverse caregiver and child populations from 0-8 years.

Methods and analysis: We will use the Arksey and O'Malley framework along with the Joanna Briggs Institute methodology for scoping reviews. We shall present our findings as per the Preferred Reporting Items for Systematic Reviews and Meta-Analyses (PRISMA) guidelines for scoping review (PRISMA-ScR). Only peer-reviewed, English-language articles from 1982 to 2022 will be included from PubMed, Web of Science, APA PsychInfo, APA PsycArticles, SocINDEX, and Google scholar databases. Reference lists of included articles will also be screened. The search strategy will be developed for each database, and search results will be imported into Rayyan. Screening will be done in two phases: i) titles and abstracts will be screened by two authors and conflicts will be resolved by mutual discussion between both or by consulting with a senior author, ii) full-texts of shortlisted studies from the first phase will then be screened using the same inclusion/exclusion criteria. A data extraction form will be developed to collate relevant information from the final list of included articles. This form will be pilot tested on the first 10 papers, and iteratively refined prior to data extraction from the remaining articles. Results will be presented in figures, tables, and a narrative summary.

Ethics and dissemination: No ethics approval needed as the review shall only use already published data. We shall publish the review in an open-access, peer-reviewed journal and disseminate through newsletters, social media pages, and presentations to relevant audiences.

Keywords: early childhood, responsive feeding, atypical development, resource-limited settings, caregiver-child interactions

For peer review only - http://bmjopen.bmj.com/site/about/guidelines.xhtml

Strengths and limitations of this study

1. To the best of our knowledge, our scoping review will be the first to understand responsive caregiving practiced in varied populations across the globe (including high- and low-middle-income countries)
2. The search strategy will include databases such as PubMed, Web of Science, APA PsychInfo, APA PsycArticles, SocINDEX, along with supplementary search in Google Scholar, and cross-referencing of the reference list of included studies.
3. We plan to use a mixed-methods approach in this review, including both quantitative and qualitative studies to include the rich data available from both types of enquiries.
4. Our study is limited by language bias due to inclusion of publications in English language only; thus, incurring a bias that certain studies published in other languages will not be included in our review.

For peer review only

For peer review only - http://bmjopen.bmj.com/site/about/guidelines.xhtml

Introduction

An estimated 250 million children in low- and middle-income countries fail to attain their developmental potential due to a range of adversities experienced during early childhood.[1] The enduring impact of these early adversities lead to major losses in human capital and productivity, thus creating a vicious cycle of intergenerational poverty. The Nurturing Care Framework launched in 2018 was prepared as a road map for caregivers, governments, civil societies, academics, and other actors.[2 3] The framework includes five components that are deemed necessary for optimizing child developmental potential. These include nutrition, health, responsive caregiving, opportunities for play, and safety and security. Considered a foundational component, responsive caregiving (RC) is defined as the ability of a caregiver to observe, notice cues (non-verbal or verbal), and respond in a timely, sensitive, and appropriate manner.[4 5] This entails a back-and-forth interaction also described as serve-and-return interaction that has been known to positively shape brain architecture. [6 7]

Responsive caregiving is bidirectional in nature and requires caregiver-child interactions that form basis of secure attachment, foster emotional bonding, and develop communication and social skills. The caregiver also provides the child with an enabling environment for learning that is safe, and allows the child to explore and practice learnings. Beginning from infancy, the importance of stimulation and responsive interaction including smiling, touching, talking, singing, reading, playing helps to develop and foster secure attachment.[8-11] In 2015, Aboud and Yousafzai undertook a systematic review that highlighted 21 interventions analyzed. These interventions, centered around responsive stimulation, and had a medium effect on children's cognitive and language development.[12] A meta-analysis in 2019 of 37 studies showed that responsive caregiving was significantly associated with children's language outcomes.[13] Studies across the world through the years have also shown that responsive caregiving in early years leads to improved physical, cognitive, and psychosocial health in children.[14-18] Moreover, data from longitudinal birth cohorts showed that responsive caregiving and early opportunities of learning were linked to higher levels of cognitive abilities during adolescence, in Brazil and South Africa.[19]

Responsive caregiving requires continuous and reciprocal interaction among the caregiver and child. Hence characteristics and contexts including maternal age, depression, income level, along with the child's development, and even gender have crucial roles.[20-23] Recently, a rapid review reported how RC was reportedly reduced during the COVID-19 pandemic, with an increase in harsh parenting due to caregiver stress, anxiety, depression.[24]

For peer review only - http://bmjopen.bmj.com/site/about/guidelines.xhtml

Even though there exists evidence that shows the differences in diverse populations, most of the studies and reviews are from the global north. As far as our current understanding extends, no comprehensive scoping review has been documented that describes the practices and outcomes of RC especially based on caregiver and child characteristics and contextual factors. Given this prevailing knowledge gap, we will follow a systematic and rigorous process, and undertake this effort to conduct an exploratory study that maps the existing literature to exclusively focus on RC practices and outcomes. Hence, a key strength of this review will be the exploration of RC practices in low- and middle-income countries and understanding their effects on child development outcomes, a context that is under-represented in global epidemiological studies.

For peer review only

For peer review only - http://bmjopen.bmj.com/site/about/guidelines.xhtml

Methods and analysis

The scoping review will be conducted using the guidance of the Arksey and O'Malley framework [25] along with the Joanna Briggs Institute method. [26] The framework will include the following steps: (1) identifying the research question/s, (2) identifying relevant studies, (3) study selection, (4) charting the data and (5) collating, summarizing and reporting the results. We will describe each stage in detail below.

Additionally, we will use the Preferred Reporting Items for Systematic Review and Meta-Analysis for scoping review (PRISMA-ScR) checklist [27] for reporting. The protocol has not been registered with PROSPERO, since it does not accept scoping review protocols. Any deviations or modifications from the protocol relevant to the study will be reported in the publication of the final report.

Stage 1: Identifying the research questions

i. What do we know about responsive caregiving (RC) practices across the world in terms of:
   a. caregiver, and child characteristics
   b. income, and location settings
   c. developmental progression over the years

ii. How does RC practices affect neurodevelopment and mental health outcomes in children (0-8 years)?

Stage 2: Identifying relevant studies

Search methods - Information sources and search strategy

A systematic search of literature published between 1982 to 2022 will be conducted among the following databases: PubMed, Web of Science, APA PsychInfo, APA PsycArticles, SocINDEX. We will use a search strategy that includes relevant terms, medical subject headings (MeSH) with appropriate Boolean operators. (See Table 1 for the pilot search strategy in PubMed.) A supplementary search in Google Scholar will also be conducted to ensure no relevant studies as missed. Additionally, cross-referencing of the reference list of the studies included will also be done. While reviews (systematic, narrative, scoping), editorials and commentaries will not be included in the current review. Detailed documentation of every search in the aforementioned databases will be done to include information on keywords used, and number of hits or retrievable studies.

For peer review only - http://bmjopen.bmj.com/site/about/guidelines.xhtml

Stage 3: Study selection based on inclusion and exclusion criteria

Table 2 summarizes the details of exclusion and inclusion criteria of studies.

Types of study

Our review will include published research articles in English language.

Population

For our review we will include studies conducted among young children (age: 0 to 8 years), and their caregivers, including biological parents, alternate caregivers (grandparent, siblings, aunts), foster, adoptive parents, etc. Since early childhood period i.e., the time from birth to eight years is a critical period in the development of many foundational skills in all areas of development, and as responsive caregiving has been considered the foundational component of nurturing care, with evidence supporting positive outcomes associated with early childhood development, we have included the same age range for children.

Concept

The studies included will describe practices of responsive caregiving hence, we will include studies that mention different forms of responsive caregiving. The most commonly used terms are responsiveness, sensitivity, warmth, caregiver-child interaction, etc. Based on preliminary literature search, additional relevant terms will be added to develop the search string.

Context

Since our aim is to synthesize a comprehensive review of responsive caregiving among diverse populations, we will include studies from low- as well high-income settings based on the socioeconomic status mentioned in the individual studies, papers from urban, rural locations, different types of families/caregivers, cultures. We will also consider studies reporting data from children with atypical development, disabilities, injuries.

Selection of studies

Search results from the databases will be imported in the Rayyan software [28], and removal of duplicate articles shall be carried out for first level screening. Based on our inclusion and exclusion criteria, articles will be screened for review by two independent authors (EL, SM) by screening of the title and abstracts through a blind review i.e., each author will be kept blind to the decision of "include" or "exclude" of articles. This will be followed by a full-text review of articles that are available. Any and all discrepancies among the authors at each step shall be discussed and resolved by senior authors (DM, PNS). At the end of the screening phase, the

For peer review only - http://bmjopen.bmj.com/site/about/guidelines.xhtml

inter-rater agreement between the reviewers will be computed using the Cohen's kappa coefficient (κ) statistic.[29]

Stage 4: Charting the data

An online form will be developed for the data extraction and will include the following details, (1) author, (2) year of publication, (3) title, (4) aims and/ or objective or research question (5) country of study, (6) study design, (7) study participants characteristics i.e., child and caregiver details such as age, sex, education, occupation, income status (8) study result/s relevant to our research question/s – including responsive caregiving practices and outcomes. The form will be initially piloted by two authors (EL, SM) with 10 included studies to ensure accuracy in terms of the study objectives and will be modified and revised as necessary. The data extraction will be done independently by the same authors, and disagreements or differences of opinions will be resolved through mutual discussion or need be by a third, senior author (DM). Through the continuous data extraction process, authors will ensure all relevant data shall be captured, and all authors are aware of the progress. All changes or modifications will be explained in the review publication.

Stage 5: Collating, summarizing, and reporting the results

For the synthesis of results of our scoping review we will prepare a PRISMA flowchart describing the search and screening of articles according to the aforementioned PRSMA-ScR checklist. This will be followed by a detailed report prepared after analyzing the extracted study characteristics. We will also include qualitative studies, hence thematic analysis of the qualitative research articles will be done and included. Therefore, based on the research questions developed, the review will include a meticulous and comprehensive understanding of RC practices and outcomes across diverse populations across the world.

Patient and public involvement

No patients involved in the protocol design.

For peer review only

Discussion

Our proposed scoping review will aim to describe responsive caregiving across global settings i.e., global south as well as global north with a special focus on whether differences exist among diverse populations. To the best of our knowledge, this review will be the first to inform regarding the practices of responsive caregiving across the world. Thus, we aim to understand characteristics of caregivers and children as well as context specific practices. We will also aim to learn about outcomes of responsive caregiving on child development during the age of 0 to eight years. We believe our review will add value to the existing knowledge by consolidating the information as well as drawing attention to the gaps in practice of responsive caregiving and early childhood development. Hence, it will hold implications for policy, practice, and research aimed at enhancing the quality of caregiving practices and subsequently positively impacting child developmental outcomes worldwide.

Ethics consideration and dissemination

Ethics approval shall not be required as this study will retrieve and synthesise data from available published literature. The scoping review will be published in an open-access, peer-reviewed journal with a high impact factor to reach larger audiences and outreach to public health researchers, social scientists, early childhood development experts, and health services researchers etc. Our review findings shall be additionally presented via our study newsletter, social media pages related to the study, as well as institute seminars and journal clubs.

Ethics statements

Patient consent for publication

Not required.

For peer review only

References

1. Lu C, Black MM, Richter LM. Risk of poor development in young children in low-income and middle-income countries: an estimation and analysis at the global, regional, and country level. *The Lancet Global Health* 2016;4(12):e916-e22.
2. Organization WH. Nurturing care for early childhood development: a framework for helping children survive and thrive to transform health and human potential, 2018.
3. Organization WH. Nurturing care for early childhood development: a framework for helping children survive and thrive to transform health and human potential. 2018
4. Landry SH, Smith KE, Swank PR, et al. The effects of a responsive parenting intervention on parent–child interactions during shared book reading. 2012;48(4):969.
5. Rasheed MA, Yousafzai AK. The development and reliability of an observational tool for assessing mother–child interactions in field studies- experience from Pakistan. *Child: care, health and development* 2015;41(6):1161-71. doi: 10.1111/cch.12287
6. Child NSCotD. Young children develop in an environment of relationships: Harvard University, Center on the Developing Child 2004.
7. Child NSCotD. The timing and quality of early experiences combine to shape brain architecture: Harvard University, Center on the Developing Child 2008.
8. Richter LM, Daelmans B, Lombardi J, et al. Investing in the foundation of sustainable development: pathways to scale up for early childhood development. 2017;389(10064):103-18.
9. Bornstein MH, Tamis-Lemonda CS. Maternal responsiveness and infant mental abilities: Specific predictive relations. *Infant Behavior and Development* 1997;20(3):283-96.
10. Tamis-LeMonda CS, Bornstein MH, Baumwell L, et al. Responsive parenting in the second year: Specific influences on children's language and play. 1996;5(4):173-83.
11. Tamis-LeMonda C, Bornstein M, Baumwell L. Maternal responsiveness and children's achievement of language milestones. *Child development* 2001;72:748-67.
12. Aboud FE, Yousafzai AK. Global health and development in early childhood. *Annual review of psychology* 2015;66:433-57.
13. Madigan S, Prime H, Graham SA, et al. Parenting Behavior and Child Language: A Meta-analysis. *Pediatrics* 2019;144(4) doi: 10.1542/peds.2018-3556 [published Online First: 2019/09/26]
14. Black MM, Walker SP, Fernald LC, et al. Early childhood development coming of age: science through the life course. 2017;389(10064):77-90.
15. Yousafzai AK, Rasheed MA, Rizvi A, et al. Effect of integrated responsive stimulation and nutrition interventions in the Lady Health Worker programme in Pakistan on child development, growth, and health outcomes: a cluster-randomised factorial effectiveness trial. 2014;384(9950):1282-93.
16. Wang K, Qi Y, Wei Q, et al. Responsive Caregiving and Opportunities for Early Learning Associated With Infant Development: Results From a Prospective Birth Cohort in China. *Frontiers in pediatrics* 2022;10:857107. doi: 10.3389/fped.2022.857107 [published Online First: 2022/07/12]
17. Hawk BN, Mccall RB, Groark CJ, et al. Caregiver sensitivity and consistency and children's prior family experience as contexts for early development within institutions. 2018;39(4):432-48.
18. Eshel N, Daelmans B, de Mello MC, et al. Responsive parenting: interventions and outcomes. *Bulletin of the World Health Organization* 2006;84(12):991-8. doi: 10.2471/blt.06.030163 [published Online First: 2007/01/24]
19. Trude AC, Richter LM, Behrman JR, et al. Effects of responsive caregiving and learning opportunities during pre-school ages on the association of early adversities and adolescent human capital: an analysis of birth cohorts in two middle-income countries. 2021;5(1):37-46.
20. Maternal depression and child development. *Paediatrics & child health* 2004;9(8):575-98. doi: 10.1093/pch/9.8.575 [published Online First: 2004/10/01]

For peer review only

For peer review only - http://bmjopen.bmj.com/site/about/guidelines.xhtml

21. Scherer E, Hagaman A, Chung E, et al. The relationship between responsive caregiving and child outcomes: evidence from direct observations of mother-child dyads in Pakistan. *BMC public health* 2019;19(1):252. doi: 10.1186/s12889-019-6571-1
22. Fielding-Gebhardt H, Warren SF, Brady NC. Child challenging behavior influences maternal mental health and relationship quality over time in fragile X syndrome. *Journal of autism and developmental disorders* 2020;50:779-97.
23. Griffith GM, Hastings RP, Petalas MA, et al. Mothers' expressed emotion towards children with autism spectrum disorder and their siblings. *Journal of intellectual disability research : JIDR* 2015;59(6):580-7. doi: 10.1111/jir.12178 [published Online First: 2014/12/19]
24. Proulx K, Lenzi-Weisbecker R, Hatch R, et al. Nurturing care during COVID-19: a rapid review of early evidence. 2022;12(6):e050417. doi: 10.1136/bmjopen-2021-050417 %J BMJ Open
25. Arksey H, O'Malley L. Scoping studies: towards a methodological framework. *International Journal of Social Research Methodology* 2005;8(1):19-32.
26. Peters MD, Godfrey CM, McInerney P, et al. The Joanna Briggs Institute reviewers' manual 2015: methodology for JBI scoping reviews. 2015
27. Tricco AC, Lillie E, Zarin W, et al. PRISMA extension for scoping reviews (PRISMA-ScR): checklist and explanation. 2018;169(7):467-73.
28. Ouzzani M, Hammady H, Fedorowicz Z, et al. Rayyan—a web and mobile app for systematic reviews. 2016;5:1-10.
29. Cohen J. A coefficient of agreement for nominal scales. *Educ Psychol Meas* 1960;20(1):37-46.

For peer review only

For peer review only - http://bmjopen.bmj.com/site/about/guidelines.xhtml

Data sharing statement: No additional data are available.

Contributors: All authors have made substantive intellectual contributions to the development of this protocol. EL, DM, PNS conceptualised the idea and the research questions. EL, SM developed and tested search terms. EL drafted the first draft of the manuscript, which was then followed by numerous iterations with critical and substantial input and appraisal from all of the authors (SM, GRB, OVS, PNS, DM). All authors approved the final version of the manuscript.

Funding: This work is supported by the DBT/Wellcome Trust India Alliance Team Science Grant [Grant No. IA/TSG/20/1/600023]. (Awarded to GRB as lead Principal investigator). Giridhara R. Babu was also supported by the DBT/Wellcome Trust India Alliance Senior Fellowship [Grant No. IA/CPHS/20/1/505278]. Prashanth N Srinivas was supported by the DBT/Wellcome Trust India Alliance CRC Grant [Grant IA/CRC/20/1/600007] awarded to him. The funders had no role in study design, methods, decision to publish, or preparation of the manuscript.

Competing interest: No competing interests were disclosed.

Provenance and peer review: Not commissioned; externally peer reviewed.

Word Count: 1727

Twitter:

Eunice Lobo @EuniceAnnLobo

Giridhara R. Babu @epigiri

Prashanth Nuggehalli Srinivas @prashanthns

Sandeep Mahapatra @S4ndeep85

Debarati Mukherjee @Debarati32

For peer review only - http://bmjopen.bmj.com/site/about/guidelines.xhtml

Table 1: Pilot search strategy in PubMed database

("responsive car*"[tiab] OR Responsiveness[tiab] OR "effective parenting"[tiab] OR "caregiver child relation*"[tiab] OR "parent child relation*"[tiab] OR "parental responsivity"[tiab] OR "maternal behaviour*"[tiab] OR "paternal behaviour*"[tiab] OR Mother-Child Relations*[MeSH] OR Mother-Child Relations*[tiab])<br>AND<br>(infant [MeSH] OR child[MeSH] OR baby[tiab] OR babies[tiab] OR toddler*[tiab] OR preschool*[tiab] OR pre school*[tiab] OR "young child*"[tiab] OR "early childhood"[tiab] OR infan*[tiab] OR newborn*[tiab] OR new-born*[tiab] OR minor*[tiab] OR kid*[tiab] OR child*[tiab] OR pediatric*[tiab] OR pediatric*[MeSH])<br>AND<br>(neurodevelop*[tiab] OR "mental health"[tiab] OR cognit*[tiab] OR “Mental health"[MeSH] OR "Child Development"[tiab] OR "infant development"[tiab] OR "Child Behavior"[MeSH] OR "Child Development"[MeSH] OR "Cognition"[MeSH])

For peer review only

For peer review only - http://bmjopen.bmj.com/site/about/guidelines.xhtml

Table 2: Eligibility criteria for study selection

Eligibility criteria	Inclusion criteria	Exclusion criteria
**Source of evidence**	Electronic databases, reference list of included studies, and Google scholar	
**Population**	Children ≤ 8 years<br>Caregivers, including biological parents, alternate caregivers (grandparent, siblings, aunts), foster, and adoptive parents.	
**Concept**	Responsive caregiving (practices, differences, stimulation, feeding, outcomes)	
**Context**	Diverse populations including caregiver, and child characteristics.	
**Publication status**	Published peer-reviewed studies	
**Language**	English	Publications in other languages<br>such as French, Chinese, etc.
**Study designs**	Primary study designs such as quantitative, qualitative, and mix-method studies<br>with human participants	Non-human/Animal studies; editorials and commentaries; other review studies such as systematic, narrative, and scoping reviews

For peer review only

For peer review only - http://bmjopen.bmj.com/site/about/guidelines.xhtml

BMJ Open

Practices and outcomes of responsive caregiving on child neurodevelopment and mental health across diverse global populations: A scoping review protocol

Journal:	*BMJ Open*
Manuscript ID	bmjopen-2023-078712.R1
Article Type:	Protocol
Date Submitted by the Author:	25-Jan-2024
Complete List of Authors:	Lobo, Eunice; Indian Institute of Public Health - Bengaluru, Public Health Foundation of India; Institute of Public Health Bengaluru<br>Mahapatra, Sandeep; Evalueserve, Bangalore, India<br>Babu, Giridhara R; Department of Population Medicine, College of Medicine, QU Health, Qatar University<br>van Schayck, Onno; Maastricht University Care and Public Health Research Institute<br>Prashanth, NS; Institute of Public Health Bengaluru<br>Mukherjee, Debarati; Indian Institute of Public Health - Bengaluru, Public Health Foundation of India
**Primary Subject Heading**:	Public health
Secondary Subject Heading:	Epidemiology, Global health, Public health
Keywords:	Community child health < PAEDIATRICS, Developmental neurology & neurodisability < PAEDIATRICS, PUBLIC HEALTH, EPIDEMIOLOGY

SCHOLARONE™
Manuscripts

Title: Practices and outcomes of responsive caregiving on child neurodevelopment and mental health across diverse global populations: A scoping review protocol

Authors: Eunice Lobo[1,2,3], Sandeep Mahapatra[4], Giridhara R. Babu[5] Onno C P van Schayck[3], Prashanth Nuggehalli Srinivas[2], Debarati Mukherjee[1]

Affiliations:
1. Indian Institute of Public Health - Bengaluru, Public Health Foundation of India, India
2. Institute of Public Health, Bengaluru, India
3. Care and Public Health Research Institute, Maastricht University, The Netherlands
4. Evalueserve, Bengaluru, India
5. Department of Population Medicine, College of Medicine, QU Health, Qatar University, Qatar

Corresponding author: Dr. Debarati Mukherjee

Address: Indian Institute of Public Health-Bengaluru, Public Health Foundation of India (PHFI), Besides Leprosy Hospital, 1st Cross, Magadi Road, Bengaluru, India – 560023
Email: debarati.mukherjee@phfi.org

For peer review only

For peer review only - http://bmjopen.bmj.com/site/about/guidelines.xhtml

Abstract

Introduction: Responsive caregiving (RC) leads to positive outcomes in children, including secure attachment with caregivers, emotional regulation, positive social interactions, and cognitive development. Through our scoping review, we aim to summarize the practices and outcomes of RC in diverse caregiver and child populations from 0-8 years.

Methods and analysis: We will use the Arksey and O'Malley framework and the Joanna Briggs Institute methodology for scoping reviews. We shall present our findings as per the Preferred Reporting Items for Systematic Reviews and Meta-Analyses (PRISMA) guidelines for scoping review (PRISMA-ScR). Only peer-reviewed, English-language articles from 1982 to 2022 will be included from PubMed, Web of Science, APA PsychInfo, APA PsycArticles, SocINDEX, and Google scholar databases. Reference lists of included articles will also be screened. The search strategy will be developed for each database, and search results will be imported into Rayyan. Screening will be done in two phases: i) titles and abstracts will be screened by two authors and conflicts will be resolved by mutual discussion between both or by consulting with a senior author, ii) full-texts of shortlisted studies from the first phase will then be screened using the same inclusion/exclusion criteria. A data extraction form will be developed to collate relevant information from the final list of included articles. This form will be pilot tested on the first 10 papers, and iteratively refined prior to data extraction from the remaining articles. Results will be presented in figures, tables, and a narrative summary.

Ethics and dissemination: No ethics approval needed as the review shall only use already published data. We shall publish the review in an open-access, peer-reviewed journal and disseminate through newsletters, social media pages, and presentations to relevant audiences.

Keywords: early childhood, responsive feeding, atypical development, resource-limited settings, caregiver-child interactions

For peer review only - http://bmjopen.bmj.com/site/about/guidelines.xhtml

Strengths and limitations of this study

1. Our study will follow the guidance of the Arksey and O'Malley framework and the Joanna Briggs Institute methodological framework for conducting a scoping review.
2. The search strategy will include databases such as PubMed, Web of Science, APA PsychInfo, APA PsycArticles, SocINDEX, along with supplementary search in Google Scholar, and cross-referencing of the reference list of included studies.
3. We plan to use a mixed-methods approach in this review, including both quantitative and qualitative studies to include the rich data available from both types of enquiries.
4. Our study is limited by language bias due to inclusion of publications in English language only; thus, incurring a bias that certain studies published in other languages will not be included in our review.

For peer review only

Introduction

An estimated 250 million children in low- and middle-income countries fail to attain their developmental potential due to a range of adversities experienced during early childhood.[1] The enduring impact of these early adversities lead to major losses in human capital and productivity, thus creating a vicious cycle of intergenerational poverty. The Nurturing Care Framework launched in 2018 was prepared as a road map for caregivers, governments, civil societies, academics, and other actors.[2, 3] The framework includes five components that are deemed necessary for optimizing child developmental potential. These include nutrition, health, responsive caregiving, opportunities for play, and safety and security. Considered a foundational component, responsive caregiving (RC) is defined as the ability of a caregiver to observe, notice cues (non-verbal or verbal), and respond in a timely, sensitive, and appropriate manner.[4, 5] This entails a back-and-forth interaction also described as serve-and-return interaction that has been known to positively shape brain architecture. [6, 7] Responsive caregiving is rooted in sensitive responsiveness (or sensitivity) as explained by Ainsworth *et al* in 1978,[8] where the caregiver responds to the child's implicit behavioural signals through appropriate, sensitive and contingent eye-gazes, facial expressions, positive affect, vocal responses, and touch. The needs of the child are responded to with empathy and timeliness while recognizing that each child has unique needs and preferences. The concept of back and forth interactions (serve-and-return) - the most efficient learning strategy for children also stems from this concept of sensitive responsiveness. Research highlights the positive impact of caregivers who consistently engage in sensitive responsiveness, contributing to fostering secure attachment,[9, 10] social and emotional well-being,[11] and positive developmental outcomes for children.[12, 13]

Responsive caregiving is bidirectional in nature and requires caregiver-child interactions that form basis of secure attachment, foster emotional bonding, and develop communication and social skills. The caregiver also provides the child with an enabling environment for learning that is safe, and allows the child to explore and practice learnings. Beginning from infancy, the importance of stimulation and responsive interaction including smiling, touching, talking, singing, reading, playing helps to develop and foster secure attachment.[14-17] For the purpose of our review we will operationalize responsive caregiving as defined by Black and Aboud in 2011 which states that responsive caregiving must include behaviour that is prompt, contingent, emotionally supportive, and developmentally appropriate (not intrusive), towards the child.[18]

For peer review only - http://bmjopen.bmj.com/site/about/guidelines.xhtml

Evidences across the years have highlighted the positive effects of responsive caregiving and child outcomes. As seen in the systematic review by Aboud and Yousafzai that highlighted 21 interventions centered around responsive stimulation, had a medium effect on children's cognitive and language development.[19] Further, a meta-analysis in 2019 of 37 studies showed that responsive caregiving was significantly associated with children's language outcomes.[12] Studies across the world through the years have also shown that responsive caregiving in early years leads to improved physical, cognitive, and psychosocial health in children.[20-24] Moreover, data from longitudinal birth cohorts showed that responsive caregiving and early opportunities of learning were linked to higher levels of cognitive abilities during adolescence, in Brazil and South Africa.[25]

Responsive caregiving requires continuous and reciprocal interaction among the caregiver and child. Hence characteristics and contexts including maternal age, depression, income level, along with the child's development, and even gender have crucial roles.[26-29] Recently, a rapid review reported how RC was reportedly reduced during the COVID-19 pandemic, with an increase in harsh parenting due to caregiver stress, anxiety, depression.[30]

Even though there exists evidence that shows the differences in diverse populations, most of the studies and reviews are from the global north. As far as our current understanding extends, no comprehensive scoping review has been documented that describes the practices and outcomes of RC especially based on caregiver and child characteristics and contextual factors. Given this prevailing knowledge gap, we will follow a systematic and rigorous process, and undertake this effort to conduct an exploratory study that maps the existing literature to exclusively focus on RC practices and outcomes. Hence, a key strength of this review will be the exploration of RC practices in low- and middle-income countries and understanding their effects on child development outcomes, a context that is under-represented in global epidemiological studies.

For peer review only

For peer review only - http://bmjopen.bmj.com/site/about/guidelines.xhtml

Methods and analysis

The scoping review will be conducted using the guidance of the Arksey and O'Malley framework [31] along with the Joanna Briggs Institute method. [32] The framework will include the following steps: (1) identifying the research question/s, (2) identifying relevant studies, (3) study selection, (4) charting the data and (5) collating, summarizing and reporting the results. We will describe each stage in detail below.

Additionally, we will use the Preferred Reporting Items for Systematic Review and Meta-Analysis for scoping review (PRISMA-ScR) checklist [33] for reporting. The protocol has not been registered with PROSPERO, since it does not accept scoping review protocols. Any deviations or modifications from the protocol relevant to the study will be reported in the publication of the final report.

Stage 1: Identifying the research questions

i. What do we know about responsive caregiving (RC) practices across the world in terms of:
   a. caregiver, and child characteristics
   b. income, and location settings
   c. cultural differences
   d. developmental progression over the years
ii. How does RC practices affect neurodevelopment and mental health outcomes in children (0-8 years)?

Stage 2: Identifying relevant studies

Search methods - Information sources and search strategy

A systematic search of literature published between 1982 to 2022 will be conducted among the following databases: PubMed, Web of Science, APA PsychInfo, APA PsycArticles, SocINDEX. We will use a search strategy that includes relevant terms, medical subject headings (MeSH) with appropriate Boolean operators. (See Table 1 for the pilot search strategy in PubMed.) A supplementary search in Google Scholar will also be conducted to ensure no relevant studies as missed. Additionally, cross-referencing of the reference list of the studies included will also be done. While reviews (systematic, narrative, scoping), editorials and commentaries will not be included in the current review. Detailed documentation of every search in the aforementioned databases will be done to include information on keywords used, and number of hits or retrievable studies.

For peer review only - http://bmjopen.bmj.com/site/about/guidelines.xhtml

Stage 3: Study selection based on inclusion and exclusion criteria

Table 2 summarizes the details of exclusion and inclusion criteria of studies.

Types of study

Our review will include published research articles in English language.

Population

For our review we will include studies conducted among young children (age: 0 to 8 years), and their caregivers, including biological parents, alternate caregivers (grandparent, siblings, aunts), foster, adoptive parents, etc. Since early childhood period i.e., the time from birth to eight years is a critical period in the development of many foundational skills in all areas of development, and as responsive caregiving has been considered the foundational component of nurturing care, with evidence supporting positive outcomes associated with early childhood development, we have included the same age range for children.

Concept

The studies included will describe practices of responsive caregiving hence, we will include studies that mention different forms of responsive caregiving. The most commonly used terms are responsiveness, sensitivity, caregiver-child interaction, etc. Based on preliminary literature search, additional relevant terms will be added to develop the search string.

Context

Since our aim is to synthesize a comprehensive review of responsive caregiving among diverse populations, we will include studies from low- as well high-income settings based on the socioeconomic status mentioned in the individual studies, papers from urban, rural locations, global south and north, different types of families/caregivers, cultures. We will also consider studies reporting data from children with atypical development, disabilities, injuries.

Selection of studies

Search results from the databases will be imported in the Rayyan software [34], and removal of duplicate articles shall be carried out for first level screening. Based on our inclusion and exclusion criteria, articles will be screened for review by two independent authors (EL, SM) by screening of the title and abstracts through a blind review i.e., each author will be kept blind to the decision of "include" or "exclude" of articles. This will be followed by a full-text review of articles that are available. Any and all discrepancies among the authors at each step shall be discussed and resolved by senior authors (DM, PNS). At the end of the screening phase, the

For peer review only - http://bmjopen.bmj.com/site/about/guidelines.xhtml

inter-rater agreement between the reviewers will be computed using the Cohen's kappa coefficient (κ) statistic.[35]

Stage 4: Charting the data

An online form will be developed for the data extraction and will include the following details, (1) author, (2) year of publication, (3) title, (4) aims and/ or objective or research question (5) country of study, (6) study design, (7) study participants characteristics i.e., child and caregiver details such as age, sex, education, occupation, income status (8) study result/s relevant to our research question/s – including responsive caregiving practices and outcomes. The form will be initially piloted by two authors (EL, SM) with 10 included studies to ensure accuracy in terms of the study objectives and will be modified and revised as necessary. The data extraction will be done independently by the same authors, and disagreements or differences of opinions will be resolved through mutual discussion or need be by a third, senior author (DM). Through the continuous data extraction process, authors will ensure all relevant data shall be captured, and all authors are aware of the progress. All changes or modifications will be explained in the review publication.

Stage 5: Collating, summarizing, and reporting the results

For the synthesis of results of our scoping review we will prepare a PRISMA flowchart describing the search and screening of articles according to the aforementioned PRSMA-ScR checklist. This will be followed by a detailed report prepared after analyzing the extracted study characteristics. We will also include qualitative studies, hence thematic analysis of the qualitative research articles will be done and included. Therefore, based on the research questions developed, the review will include a meticulous and comprehensive understanding of RC practices and outcomes across diverse populations across the world.

Patient and public involvement

No patients involved in the protocol design.

For peer review only - http://bmjopen.bmj.com/site/about/guidelines.xhtml

Discussion

Our proposed scoping review will aim to describe responsive caregiving across global settings i.e., global south as well as global north with a special focus on whether differences exist among diverse populations. To the best of our knowledge, this review will be the first to inform regarding the practices of responsive caregiving across the world. Thus, we aim to understand characteristics of caregivers and children as well as context specific practices. We will also aim to learn about outcomes of responsive caregiving on child development during the age of 0 to eight years. We believe our review will add value to the existing knowledge by consolidating the information as well as drawing attention to the gaps in practice of responsive caregiving and early childhood development. Hence, it will hold implications for policy, practice, and research aimed at enhancing the quality of caregiving practices and subsequently positively impacting child developmental outcomes worldwide.

Ethics consideration and dissemination

Ethics approval shall not be required as this study will retrieve and synthesise data from available published literature. The scoping review will be published in an open-access, peer-reviewed journal with a high impact factor to reach larger audiences and outreach to public health researchers, social scientists, early childhood development experts, and health services researchers etc. Our review findings shall be additionally presented via our study newsletter, social media pages related to the study, as well as institute seminars and journal clubs.

Ethics statements

Patient consent for publication

Not required.

For peer review only - http://bmjopen.bmj.com/site/about/guidelines.xhtml

References

1. Lu C, Black MM, Richter LM. Risk of poor development in young children in low-income and middle-income countries: an estimation and analysis at the global, regional, and country level. The Lancet Global Health. 2016;4(12):e916-e22.
2. Organization WH. Nurturing care for early childhood development: a framework for helping children survive and thrive to transform health and human potential. 2018. Report No.: 924151406X.
3. Organization WH. Nurturing care for early childhood development: a framework for helping children survive and thrive to transform health and human potential. 2018.
4. Landry SH, Smith KE, Swank PR, Zucker T, Crawford AD, Solari EFJDp. The effects of a responsive parenting intervention on parent–child interactions during shared book reading. 2012;48(4):969.
5. Rasheed MA, Yousafzai AK. The development and reliability of an observational tool for assessing mother–child interactions in field studies- experience from Pakistan. Child: care, health and development. 2015;41(6):1161-71.
6. Child NSCotD. Young children develop in an environment of relationships: Harvard University, Center on the Developing Child; 2004.
7. Child NSCotD. The timing and quality of early experiences combine to shape brain architecture: Harvard University, Center on the Developing Child; 2008.
8. Ainsworth MDS, Blehar MC, Waters E, Wall S. Patterns of attachment: A psychological study of the strange situation. Oxford, England: Lawrence Erlbaum; 1978. xviii, 391-xviii, p.
9. Madigan S, Deneault A, Duschinsky R, Bakermans-Kranenburg M, Schuengel C, van IJzendoorn Marinus H. Parent sensitivity and child attachment: a metaanalysis revisited. Psychol Bull. 2023;149(1-2):99-132.
10. Dunst CJ, Kassow DZ. Caregiver sensitivity, contingent social responsiveness, and secure infant attachment. The Journal of Early and Intensive Behavioral Intervention. 2008;5:40-56.
11. Ainsworth MDS, Bell SM, Stayton DJ. Infant–Mother Attachment and Social Development:'Socialisation'as a Product of Reciprocal Responsiveness to Signals. Becoming a person: Routledge; 2013. p. 30-55.
12. Madigan S, Prime H, Graham SA, Rodrigues M, Anderson N, Khoury J, et al. Parenting Behavior and Child Language: A Meta-analysis. Pediatrics. 2019;144(4).
13. Treyvaud K, Doyle LW, Lee KJ, Ure A, Inder TE, Hunt RW, et al. Parenting behavior at 2 years predicts school-age performance at 7 years in very preterm children. Journal of Child Psychology and Psychiatry. 2016;57(7):814-21.
14. Richter LM, Daelmans B, Lombardi J, Heymann J, Boo FL, Behrman JR, et al. Investing in the foundation of sustainable development: pathways to scale up for early childhood development. 2017;389(10064):103-18.
15. Bornstein MH, Tamis-Lemonda CS. Maternal responsiveness and infant mental abilities: Specific predictive relations. Infant Behavior and Development. 1997;20(3):283-96.
16. Tamis-LeMonda CS, Bornstein MH, Baumwell L, Melstein Damast AJED, Research PAIJo, Practice. Responsive parenting in the second year: Specific influences on children's language and play. 1996;5(4):173-83.
17. Tamis-LeMonda C, Bornstein M, Baumwell L. Maternal responsiveness and children's achievement of language milestones. Child development. 2001;72:748-67.
18. Black MM, Aboud FE. Responsive feeding is embedded in a theoretical framework of responsive parenting. The Journal of nutrition. 2011;141(3):490-4.
19. Aboud FE, Yousafzai AK. Global health and development in early childhood. Annual review of psychology. 2015;66:433-57.
20. Black MM, Walker SP, Fernald LC, Andersen CT, DiGirolamo AM, Lu C, et al. Early childhood development coming of age: science through the life course. 2017;389(10064):77-90.
21. Yousafzai AK, Rasheed MA, Rizvi A, Armstrong R, Bhutta ZAJTL. Effect of integrated responsive stimulation and nutrition interventions in the Lady Health Worker programme in Pakistan

For peer review only - http://bmjopen.bmj.com/site/about/guidelines.xhtml

on child development, growth, and health outcomes: a cluster-randomised factorial effectiveness trial. 2014;384(9950):1282-93.
22. Wang K, Qi Y, Wei Q, Shi Y, Zhang Y, Shi H. Responsive Caregiving and Opportunities for Early Learning Associated With Infant Development: Results From a Prospective Birth Cohort in China. Frontiers in pediatrics. 2022;10:857107.
23. Hawk BN, Mccall RB, Groark CJ, Muhamedrahimov RJ, Palmov OI, Nikiforova NVJImhj. Caregiver sensitivity and consistency and children's prior family experience as contexts for early development within institutions. 2018;39(4):432-48.
24. Eshel N, Daelmans B, de Mello MC, Martines J. Responsive parenting: interventions and outcomes. Bulletin of the World Health Organization. 2006;84(12):991-8.
25. Trude AC, Richter LM, Behrman JR, Stein AD, Menezes AM, Black MMJTLC, et al. Effects of responsive caregiving and learning opportunities during pre-school ages on the association of early adversities and adolescent human capital: an analysis of birth cohorts in two middle-income countries. 2021;5(1):37-46.
26. Maternal depression and child development. Paediatr Child Health. 2004;9(8):575-98.
27. Scherer E, Hagaman A, Chung E, Rahman A, O'Donnell K, Maselko J. The relationship between responsive caregiving and child outcomes: evidence from direct observations of mother-child dyads in Pakistan. BMC Public Health. 2019;19(1):252.
28. Fielding-Gebhardt H, Warren SF, Brady NC. Child challenging behavior influences maternal mental health and relationship quality over time in fragile X syndrome. Journal of autism and developmental disorders. 2020;50:779-97.
29. Griffith GM, Hastings RP, Petalas MA, Lloyd TJ. Mothers' expressed emotion towards children with autism spectrum disorder and their siblings. Journal of intellectual disability research : JIDR. 2015;59(6):580-7.
30. Proulx K, Lenzi-Weisbecker R, Hatch R, Hackett K, Omoeva C, Cavallera V, et al. Nurturing care during COVID-19: a rapid review of early evidence. 2022;12(6):e050417.
31. Arksey H, O'Malley L. Scoping studies: towards a methodological framework. International Journal of Social Research Methodology 2005;8(1):19-32.
32. Peters MD, Godfrey CM, McInerney P, Soares CB, Khalil H, Parker D. The Joanna Briggs Institute reviewers' manual 2015: methodology for JBI scoping reviews. 2015.
33. Tricco AC, Lillie E, Zarin W, O'Brien KK, Colquhoun H, Levac D, et al. PRISMA extension for scoping reviews (PRISMA-ScR): checklist and explanation. 2018;169(7):467-73.
34. Ouzzani M, Hammady H, Fedorowicz Z, Elmagarmid AJSr. Rayyan—a web and mobile app for systematic reviews. 2016;5:1-10.
35. Cohen J. A coefficient of agreement for nominal scales. Educ Psychol Meas. 1960;20(1):37-46.

For peer review only

For peer review only - http://bmjopen.bmj.com/site/about/guidelines.xhtml

For peer review only

For peer review only - http://bmjopen.bmj.com/site/about/guidelines.xhtml

Data sharing statement: No additional data are available.

Contributors: All authors have made substantive intellectual contributions to the development of this protocol. EL, DM, PNS conceptualised the idea and the research questions. EL, SM developed and tested search terms. EL drafted the first draft of the manuscript, which was then followed by numerous iterations with critical and substantial input and appraisal from all of the authors (SM, GRB, OVS, PNS, DM). All authors approved the final version of the manuscript.

Funding: This work is supported by the DBT/Wellcome Trust India Alliance Team Science Grant [Grant No. IA/TSG/20/1/600023] (Awarded to GRB as lead Principal investigator). Prashanth N Srinivas was supported by the DBT/Wellcome Trust India Alliance CRC Grant [Grant IA/CRC/20/1/600007] awarded to him. The funders had no role in study design, methods, decision to publish, or preparation of the manuscript.

Competing interest: No competing interests were disclosed.

Provenance and peer review: Not commissioned; externally peer reviewed.

Word Count: 1907

Twitter:

Eunice Lobo @EuniceAnnLobo

Giridhara R. Babu @epigiri

Prashanth Nuggehalli Srinivas @prashanthns

Sandeep Mahapatra @S4ndeep85

Debarati Mukherjee @Debarati32

For peer review only

For peer review only - http://bmjopen.bmj.com/site/about/guidelines.xhtml

Table 1: Pilot search strategy in PubMed database

("responsive car*"[tiab] OR Responsiveness[tiab] OR "effective parenting"[tiab] OR "caregiver child relation*"[tiab] OR "parent child relation*"[tiab] OR "parental responsivity"[tiab] OR "maternal behaviour*"[tiab] OR "paternal behaviour*"[tiab] OR Mother-Child Relations*[MeSH] OR Mother-Child Relations*[tiab])

AND

(infant [MeSH] OR child[MeSH] OR baby[tiab] OR babies[tiab] OR toddler*[tiab] OR preschool*[tiab] OR pre school*[tiab] OR "young child*"[tiab] OR "early childhood"[tiab] OR infan*[tiab] OR newborn*[tiab] OR new-born*[tiab] OR minor*[tiab] OR kid*[tiab] OR child*[tiab] OR pediatric*[tiab] OR pediatric*[MeSH])

AND

(neurodevelop*[tiab] OR "mental health"[tiab] OR cognit*[tiab] OR “Mental health"[MeSH] OR "Child Development"[tiab] OR "infant development"[tiab] OR "Child Behavior"[MeSH] OR "Child Development"[MeSH] OR "Cognition"[MeSH])

For peer review only

For peer review only - http://bmjopen.bmj.com/site/about/guidelines.xhtml

359 **Table 2: Eligibility criteria for study selection**

Eligibility criteria	Inclusion criteria	Exclusion criteria
**Source of evidence**	Electronic databases, reference list of included studies, and Google scholar	
**Population**	Children ≤ 8 years<br>Caregivers, including biological parents, alternate caregivers (grandparent, siblings, aunts), foster, day-care providers, and adoptive parents.	
**Concept**	Responsive caregiving (practices, differences, stimulation, feeding, outcomes)	
**Context**	Diverse populations including caregiver, and child characteristics, income, location, cultural differences	
**Publication status**	Published peer-reviewed studies	
**Language**	English	Publications in other languages<br>such as French, Chinese, etc.
**Study designs**	Primary study designs such as quantitative including observational, randomized controlled trials, interventions, qualitative, and mix-method studies<br>with human participants	Non-human/Animal studies; editorials and commentaries; other review studies such as systematic, narrative, and scoping reviews

360

For peer review only - http://bmjopen.bmj.com/site/about/guidelines.xhtml

Preferred Reporting Items for Systematic reviews and Meta-Analyses extension for Scoping Reviews (PRISMA-ScR) Checklist

SECTION	ITEM	PRISMA-ScR CHECKLIST ITEM	REPORTED ON PAGE #
**TITLE**			
Title	1	Identify the report as a scoping review.	1
**ABSTRACT**			
Structured summary	2	Provide a structured summary that includes (as applicable): background, objectives, eligibility criteria, sources of evidence, charting methods, results, and conclusions that relate to the review questions and objectives.	2
**INTRODUCTION**			
Rationale	3	Describe the rationale for the review in the context of what is already known. Explain why the review questions/objectives lend themselves to a scoping review approach.	5
Objectives	4	Provide an explicit statement of the questions and objectives being addressed with reference to their key elements (e.g., population or participants, concepts, and context) or other relevant key elements used to conceptualize the review questions and/or objectives.	6,7
**METHODS**			
Protocol and registration	5	Indicate whether a review protocol exists; state if and where it can be accessed (e.g., a Web address); and if available, provide registration information, including the registration number.	NA
Eligibility criteria	6	Specify characteristics of the sources of evidence used as eligibility criteria (e.g., years considered, language, and publication status), and provide a rationale.	7; Table 2
Information sources*	7	Describe all information sources in the search (e.g., databases with dates of coverage and contact with authors to identify additional sources), as well as the date the most recent search was executed.	6

For peer review only

SECTION	ITEM	PRISMA-ScR CHECKLIST ITEM	REPORTED ON PAGE #
Search	8	Present the full electronic search strategy for at least 1 database, including any limits used, such that it could be repeated.	Table 1
Selection of sources of evidence†	9	State the process for selecting sources of evidence (i.e., screening and eligibility) included in the scoping review.	6,7,8
Data charting process‡	10	Describe the methods of charting data from the included sources of evidence (e.g., calibrated forms or forms that have been tested by the team before their use, and whether data charting was done independently or in duplicate) and any processes for obtaining and confirming data from investigators.	8
Data items	11	List and define all variables for which data were sought and any assumptions and simplifications made.	8
Critical appraisal of individual sources of evidence§	12	If done, provide a rationale for conducting a critical appraisal of included sources of evidence; describe the methods used and how this information was used in any data synthesis (if appropriate).	NA
Synthesis of results	13	Describe the methods of handling and summarizing the data that were charted.	8
		**RESULTS**	
Selection of sources of evidence	14	Give numbers of sources of evidence screened, assessed for eligibility, and included in the review, with reasons for exclusions at each stage, ideally using a flow diagram.	NA
Characteristics of sources of evidence	15	For each source of evidence, present characteristics for which data were charted and provide the citations.	NA
Critical appraisal within sources of evidence	16	If done, present data on critical appraisal of included sources of evidence (see item 12).	NA
Results of individual sources of evidence	17	For each included source of evidence, present the relevant data that were charted that relate to the review questions and objectives.	NA
Synthesis of results	18	Summarize and/or present the charting results as they relate to the review questions and objectives.	NA

For peer review only - http://bmjopen.bmj.com/site/about/guidelines.xhtml

SECTION	ITEM	PRISMA-ScR CHECKLIST ITEM	REPORTED ON PAGE #
**DISCUSSION**			
Summary of evidence	19	Summarize the main results (including an overview of concepts, themes, and types of evidence available), link to the review questions and objectives, and consider the relevance to key groups.	NA
Limitations	20	Discuss the limitations of the scoping review process.	NA
Conclusions	21	Provide a general interpretation of the results with respect to the review questions and objectives, as well as potential implications and/or next steps.	NA
**FUNDING**			
Funding	22	Describe sources of funding for the included sources of evidence, as well as sources of funding for the scoping review. Describe the role of the funders of the scoping review.	12

JBI = Joanna Briggs Institute; PRISMA-ScR = Preferred Reporting Items for Systematic reviews and Meta-Analyses extension for Scoping Reviews.
* Where *sources of evidence* (see second footnote) are compiled from, such as bibliographic databases, social media platforms, and Web sites.
† A more inclusive/heterogeneous term used to account for the different types of evidence or data sources (e.g., quantitative and/or qualitative research, expert opinion, and policy documents) that may be eligible in a scoping review as opposed to only studies. This is not to be confused with *information sources* (see first footnote).
‡ The frameworks by Arksey and O'Malley (6) and Levac and colleagues (7) and the JBI guidance (4, 5) refer to the process of data extraction in a scoping review as data charting.
§ The process of systematically examining research evidence to assess its validity, results, and relevance before using it to inform a decision. This term is used for items 12 and 19 instead of "risk of bias" (which is more applicable to systematic reviews of interventions) to include and acknowledge the various sources of evidence that may be used in a scoping review (e.g., quantitative and/or qualitative research, expert opinion, and policy document).

From: Tricco AC, Lillie E, Zarin W, O'Brien KK, Colquhoun H, Levac D, et al. PRISMA Extension for Scoping Reviews (PRISMAScR): Checklist and Explanation. Ann Intern Med. 2018;169:467–473. doi: 10.7326/M18-0850.

For peer review only - http://bmjopen.bmj.com/site/about/guidelines.xhtml
